# Genome Annotation for Pinkcreek, a C1 Subcluster Mycobacteriophage from New Orleans, Louisiana

**DOI:** 10.1128/mra.00576-22

**Published:** 2022-08-18

**Authors:** Christine A. Byrum, Maximiliano F. Flota, Jackson A. Derrick, Steven Grant Dixon, Andre S. Gagliano, Halee S. Gibson, Paige A. Loose, Ashley Montero, Jessica L. Mugford, Emily E. Mullner, Briana C. Pope, Christopher A. Korey

**Affiliations:** a Department of Biology, College of Charleston, Charleston, South Carolina, USA; Loyola University Chicago

## Abstract

The mycobacteriophage Pinkcreek (C1 subcluster) was extracted from soil collected on the Dr. Norman C. Francis Parkway Bike Trail in New Orleans, Louisiana. It is a member of the family *Myoviridae* and infects Mycobacterium smegmatis mc^2^155. The Pinkcreek genome is 153,184 bp and contains 216 predicted protein-coding genes, 29 tRNAs, and 1 transfer-messenger RNA.

## ANNOUNCEMENT

In a broad effort to better characterize viral diversity and evolution, the bacteriophage Pinkcreek was extracted from soil gathered on the Dr. Norman C. Francis Parkway Bike Trail in New Orleans, Louisiana (29.9619N, 90.1013W), during fall 2018 (Table 1). This project was sponsored by the Howard Hughes Medical Institute (HHMI) Science Education Alliance-Phage Hunters Advancing Genomics and Evolutionary Science (SEA-PHAGES) program (1), and Pinkcreek was isolated by direct plating followed by two cycles of purification/amplification using 7H9 top agar containing Mycobacterium smegmatis mc^2^155 at 37°C, in accordance with the SEA-PHAGES Discovery Guide (2).

**TABLE 1:** Characteristics of the Pinkcreek bacteriophage

Parameter	Pinkcreek data
GenBank accession no.	MZ958745
SRA accession no.	SRX13720608
Collection site	New Orleans, Louisiana, USA
Collection site coordinates	29.9619N, 90.1013W
Isolation host	Mycobacterium smegmatis mc^2^155
Genome size (bp)	153,184
Coverage (×)	353
GC content (%)	64.7
No. of predicted protein-coding genes	216
No. of tRNAs	29
No. of tmRNAs	1
Morphotype	*Myoviridae*
Subcluster	C1
Predicted protein-coding genes (phams) unique to and conserved in all C1 subcluster members^*a*^	4, 5, 13, 23, 25, 29, 44, 50, 51, 54, 55 (helix-turn-helix DNA binding domain protein), 57, 59, 62, 68, 69, 82, 94, 98, 104, 107, 112 (acetyltransferase),129, 132–134, 138, 139, 142, 206, 212–214, 231 (membrane protein), 234–237, 239, 240 (serine/threonine kinase), 241 (HNH endonuclease), 243 (PurA-like adenylosuccinate synthetase)

a Based on data available in Phamerator on 1 June 2022 (19). Sequences with known predicted functions are indicated in parentheses.

To sequence the genome, DNA was extracted from high-titer lysates using the Promega Wizard DNA cleanup system, and a sequencing library was prepared with the NEBNext Ultra II library prep kit (v3 reagents). Pittsburgh Bacteriophage Institute sequenced the DNA on an Illumina MiSeq system (MiSeq reagent kit v3) (3), and 382,828 single-end reads (150 bp) were obtained (coverage, 353×; average Phred score, 37.29). Raw reads were assembled *de novo* into a single contig using Newbler v2.9 (4), and editing and finishing were performed with Consed v29.0 (3, 5). Lack of read buildups (detected using PAUSE [https://cpt.tamu.edu/computer-dresourcs/pause]) indicated that the 153,184-bp genome (GC content, 64.6%) is circularly permuted. AceUtil (http://phagesdb.org/AceUtil) was utilized to check for sequence discrepancies and low-coverage sites. Further details were described by Russell (3).

Pinkcreek was annotated using the PECAAN workflow tool (6), with start sites determined using GeneMark v2.5 (7), GLIMMER v3.02 (8), and Starterator v1.1 (9); functional calls were made with HHpred (10), BLASTp v2.13.0+ (11), TOPCONS v2 (12), TMHMM2 (https://services.healthtech.dtu.dk/service.php?TMHMM-2.0), SOSUI v1.11 (13), and the NCBI Conserved Domain Database (CDD) (14), while tRNAs and transfer-messenger RNAs (tmRNAs) were identified using tRNAscan-SE v3.0 (15) and ARAGORN v1.2.38 (16). Parameters and databases used by PECAAN for the HHpred, BLASTp, and CDD searches are summarized at https://seaphages.org/forums/topic/5398. Other programs utilized default parameters. After annotation, data were transferred to DNA Master v5.22.2 (https://phagesdb.org/DNAMaster).

Based on nucleotide sequence similarities, phages are assigned to clusters sharing nucleotide sequence similarity of >50% (17) and/or gene content similarity of ≥35% (18). Pinkcreek, a C cluster/C1 subcluster member, is a lytic mycobacteriophage with *Myoviridae* morphology and a genome containing 216 predicted protein-coding genes (46 with assigned putative functions), 29 tRNAs, and 1 tmRNA. To compare Pinkcreek’s genome to those of other actinobacteriophages, the program Phamerator (19) was used. Phamerator generates a map of the genome and, by selecting a single gene, users can access a pulldown menu listing all actinobacteriophage clusters (and cluster members) with the same pham (homologous protein-coding genes sharing ≥32.5% identity). Pinkcreek’s genome has 42 phams that are conserved in all C1 subcluster members (*n* = 160) but are absent in other actinobacteriophages (Table 1). The genome also contains an orpham (gp135), a tandem duplication (gp100/gp101), and a rare pham (gp12) encountered in only three other C1 subcluster members, namely, HyRo (GenBank accession number KT281790), Shifa (GenBank accession number MT889395), and Stubby (GenBank accession number MK450423). All genes are transcribed on the forward strand except gp39 to gp41, gp137 to gp138, and gp171 to gp172, and whole-genome BLASTn alignments (11) revealed that the C1 subcluster member Alice (GenBank accession number JF704092) (99.58% identity and 97% coverage) is most similar to Pinkcreek. Other similar C1 subcluster members (≥99% identity and ≥92% coverage) include Blackbrain (GenBank accession number MK878897), Grungle (GenBank accession number MN062707), Koguma (GenBank accession number MF919513), LinStu (GenBank accession number JN412592), and Sauce (GenBank accession number NC_054722). Four tRNAs present in most of these phages are absent in Pinkcreek (would normally occur between base 92747 and base 92767).

### Data availability.

GenBank and SRA accession numbers are presented in Table 1.
